# Cumulative Incidence of Revision for a Balanced Knee System at a Mean 8-Year Follow-Up: A Retrospective Review of 500 Consecutive Total Knee Arthroplasties

**DOI:** 10.1155/2019/9580586

**Published:** 2019-06-02

**Authors:** Michael H. Bourne, Tony L. Miller, E. Marc Mariani

**Affiliations:** Salt Lake Orthopaedic Clinic, St. Mark's Hospital, Salt Lake City, Utah, USA

## Abstract

**Purpose:**

The primary purpose of this study was to evaluate mid-term survival of a Balanced Knee System in the first 500 total knee arthroplasty (TKA) cases using a fully cemented, posterior stabilized TKA at a high-volume private practice.

**Patients and Methods:**

In this IRB approved retrospective cohort study, data were extracted from a surgical registry at a high-volume orthopaedic practice for the first 500 total knee arthroplasty (TKA) cases performed using the Balanced Knee® System (BKS, Ortho Development®, Draper, Utah, USA). Procedures were performed between June 2000 and September 2003 by one of two orthopaedic surgeons. Follow-up was performed at 6 weeks, 6 months, 1 year, 5 years, and 10 years. 48 patients (9.6%) were considered lost to follow-up. A competing risk analysis was performed to evaluate the cumulative incidence of revision while accounting for the competing risk of death. In the model, failure was defined as revision of any BKS component. Those who failed prior to two years remained in the analysis.

**Results:**

The mean age of the population was 69 years (range: 40–94) and 73% were female. The cumulative incidence of revision of any component was approximately 1% at a mean 8-year follow-up (range: 0.11–14.1 years) when accounting for the competing risk of death. When considering all those lost to follow-up as failures, the cumulative incidence of failure at 8 years was approximately 10%.

**Conclusion:**

Based on the results of the current study, a posterior stabilized primary TKA, implanted using a flexion and extension gap balancing technique, had excellent survivorship and outcomes at a mean 8-year follow-up.

## 1. Introduction

Total knee arthroplasty (TKA) is one of the most common surgical procedures for restoring physical function and mitigating pain associated with arthritic knees, and the frequency of TKA is expected to rise [1, 2]. When performing this procedure, one common technique is gap balancing [3]. This technique involves a step-by-step approach to ensure equal flexion and extension gaps with equalization of soft tissue tension [3]. Proponents of this technique note that the equalization of soft tissue tension, as well as the creation of equal flexion and extension gaps, improves the function and longevity of TKA [4–8].

However, this technique presents with some limitations. In a recent review of surgical techniques for TKA, Sheth et al. [3] reported that the gap balancing technique could result in raising of the joint line, mid-flexion instability, irregularities in femoral rotation, and excessive flexion gap when performed in posterior stabilized TKA [9]. Given these potential pitfalls, continued postmarket clinical follow-up for gap balancing systems is needed.

The purpose of this study was to evaluate mid-term survival of a Balanced Knee System in the first 500 total knee arthroplasty (TKA) cases using a fully cemented, posterior stabilized TKA at a high-volume private practice. Additionally, we sought to evaluate the frequency of postoperative complications associated with the surgical procedure, the reasons for revision, radiographic outcomes, and differences in Knee Society Scores and arc of motion from pre- to last follow-up visits.

## 2. Materials and Methods

**Figure 1:**
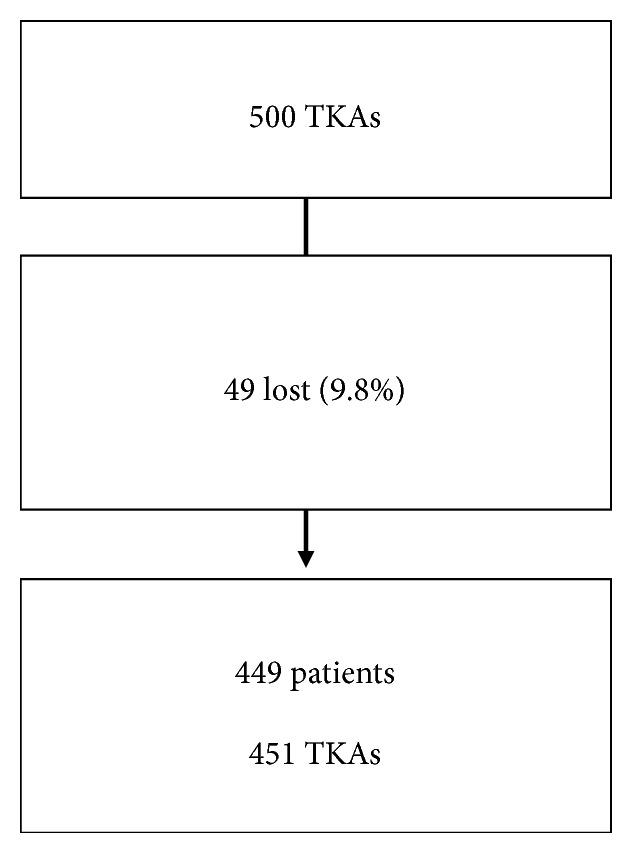
Flow diagram demonstrating patient attrition.

After obtaining approval from the institutional review board (IRB# 0114), data were extracted from a surgical registry at a high-volume orthopaedic practice for the first 500 total knee arthroplasty (TKA) cases performed using the Balanced Knee® System (BKS, Ortho Development®, Draper, Utah, USA). Posterior stabilized, fully cemented implants were used in all knees. The procedures were performed between June 2000 and September 2003 by one of two high-volume, private practice, orthopaedic surgeons. Follow-up was performed during routine office visits or phone call follow-up at 6 weeks, 6 months, 1 year, 5 years, and 10 years. A minimum two-year follow-up was required for each patient in order to be included in the analysis. If patients did not have a minimum two-year follow-up, public death records were referenced to determine if a date of death was present. Patients without a minimum two-year follow-up, who had not experienced a known postoperative complication before, and without a confirmed death record were considered “lost to follow-up”. Of the 500 TKAs, 48 patients (9.6%) were considered lost to follow-up (Figure 1).

### 2.1. Surgical Procedure

The surgical procedure was standardized between the two surgeons, both performing a total knee arthroplasty utilizing the medial retinacular approach, tourniquet control, and general anesthetic and femoral nerve block. Gap balancing was performed using both bony resection and soft tissue balancing. The bony portion of the balancing technique used an intramedullary guide on the femur with the distal cut at 5° valgus, and an extramedullary tibial guide was used with the proximal cut perpendicular to the longitudinal axis of the tibia in the coronal plane and following the normal tibial posterior slope of 3–5 degrees in the sagittal plane. A recent review of the literature suggests an ideal posterior slope of 0–7 degrees [10]. The distal femoral cut in conjunction with the proximal tibial resection determined the extension gap.

**Figure 2:**
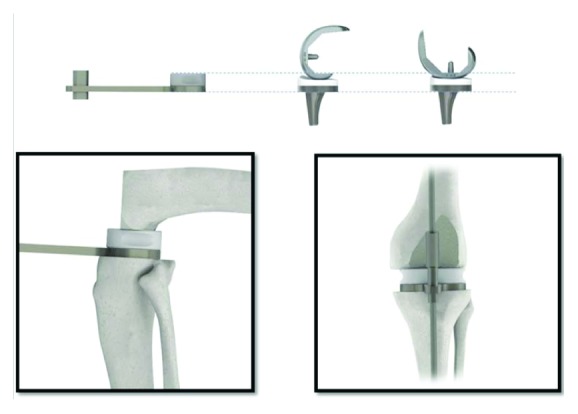
The spacer block system provided in the instrumentation of the Balanced Knee System used in this study.

The anterior and posterior femoral resections were made in line with the epicondylar axis or perpendicular to Whiteside's line. The posterior femoral cut combined with the proximal tibial surface defined the flexion gap. Posterior femoral osteophytes as well as medial and lateral femoral and tibial osteophytes were resected. Soft tissue balancing was next performed followed by adjusted bony cuts to create symmetric rectangular flexion and extension gaps. A composite spacer block (replicating the total thickness of the tibial component plus the femoral component plus the polyethylene spacer) was utilized during the process to aid in the extent of soft tissue releases and additional boney cuts to ultimately create medial-lateral soft tissue balancing with equalization of the flexion and extension gaps (Figure 2). With this system, the spacer blocks increase by 1mm increments from 5 to 14 mm, with additional blocks of 16, 18, and 20 mm if needed. This allows for precise measuring of the flexion-extension gaps for placement of the appropriate tibial insert. The tibial inserts range in size from 7 to 14mm, also increasing by 1mm each, followed by 16,18, and 20mm inserts.

After the initial bone cuts and equalization of the soft tissues, if the flexion gap was tighter than the extension gap, the flexion gap was enlarged by downsizing the femoral component, as the additional bone removed in downsizing was taken from the posterior femoral condyles. In cases where the extension gap was tighter than the flexion gap, an appropriate amount of bone was taken from the distal femur. When gaps are equally too tight in both flexion and extension, additional tibial resection was performed to enlarge the flexion and extension gaps equally.

Only after the flexion and extension gaps were equalized and soft tissues balanced, were finishing cuts (notch and chamfers) completed on the femur. The center tibial component rotation is determined by aligning the center of the component facing the medial third of the tibial tubercle which usually aligns the anterior edge of the tibial component parallel with the anterior edge of the tibia. A trial reduction was then performed ensuring stability throughout a full range of motion.

Both surgeons utilized a layered closure of absorbable suture with a final subcuticular closure using a nonabsorbable suture. Patients averaged three days postoperative hospitalization with a return to home or an extended care facility. Both physicians used Coumadin for deep vein thrombosis prophylaxis as well as perioperative intermittent compression boots and compression hose. Postoperative physical therapy and continuous passive motion machines were employed as part of the standard care-pathways.

### 2.2. Outcome Measures

Revisions and surgical complications were collected from the surgical database and verified by review of the electronic medical records. Additionally, manual chart review was performed on a random sample of 20% of the population (91/451) in order to verify the outcomes from the database. Of these, charts were unavailable for 7/451 (1.6%) knees. The findings from the review were consistent with the database results, and no further review was performed. A failure of the implant was defined as any post-arthroplasty removal of one or more implant, or polyethylene, components for any reason. A complication was defined as an untoward surgical or post-arthroplasty event that did not require removal of an implant component, but that was directly related to the arthroplasty procedure.

Hip to ankle radiographs were obtained prior to surgery to assess lower extremity alignment and after TKA to determine anatomical axis. Of the 406 postoperative X-rays, with minimum two-year follow-up, available for review, 344 (85%) were completed in a standing position, 35 (9%) were completed in a recumbent position, and 27 (7%) were missing this information. Radiographs were evaluated using the knee society roentgenographic rating system [11].

As part of routine care, the Knee Society Clinical Rating System (KS) was used [12]. The KS score is composed of both a knee score and a functional score. The scores are reported on a scale of 0–100 with higher scores indicating better outcomes. Lee et al. [13] have recently reported a minimal clinically important difference for the KS score at approximately 6 points for both the knee and functional scores with 95% confidence intervals of roughly 4–8 points.

Patient range of motion (ROM) was assessed in the office by the surgeon, using a standard goniometer with the patient in the supine position, and is reported as the arc of motion (complete extension to full flexion).

### 2.3. Statistical Analysis

Given the large proportion of patients expired (26%, 116/451) over the course of the study period, a competing risk analysis, using the methods of Fine & Gray, was performed to evaluate the cumulative incidence of revision while accounting for the competing risk of death [14, 15]. In the model, failure was defined as revision of any BKS component. A worst-case analysis was also performed where failure was defined as revision or lost to follow-up. As there was approximately 11% missing data for KSS and arc of motion outcome variables, imputations were performed using the multivariate imputation by chained equations (MICE) method of multiple multivariate imputation [16–18]. A simple linear generalized estimating equation (GEE) regression analysis with an independent correlation matrix was then performed to evaluate the difference between pre- and last follow-up visits in the KS knee score, KS functional score, and arc of motion. Model fit was assessed using the quasi-likelihood independence model criterion [19]. The minimum clinically important difference (MCID) was considered as a change of 6 points on the KS scores as described by Lee et al. [13]. Given the high mortality rate, and in order to better understand the survival free from death, a subanalysis was performed using a Kaplan–Meier analysis with death as failure. Additionally, the proportion of expired patients was dichotomized by age ≥80 vs <80 and evaluated using a chi-square analysis. Statistical analyses were performed using Stata/IC version 15.1 (College Station, Texas, USA). Significance was assessed at p<0.05.

## 3. Results

The mean age of the population was 69 years (range: 40–94) with 73% being female (Table 1). Osteoarthritis was the primary diagnosis for 95% (428/451) of the cases (Table 1). The mean preoperative coronal alignment was 5° (range: 1°–16°).

**Figure 3:**
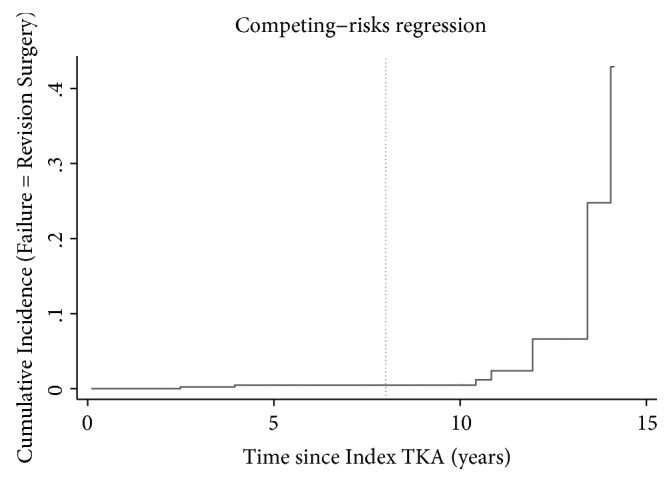
The cumulative incidence curve with failure defined as revision of any component.

**Figure 4:**
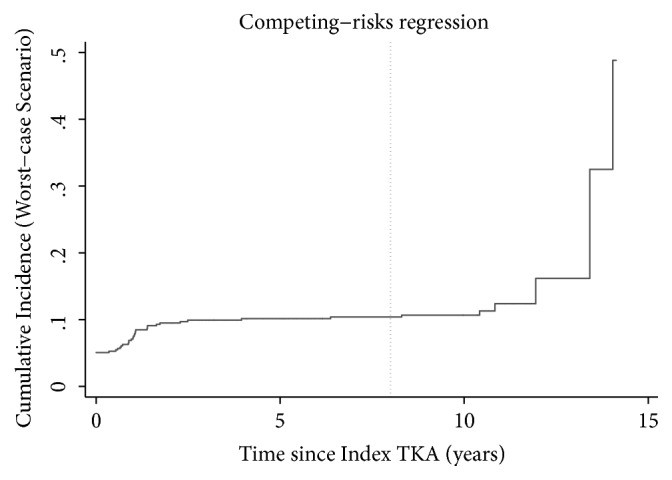
The cumulative incidence curve for the worst-case scenario with failure defined as revision of any component or lost to follow-up.

**Figure 5:**
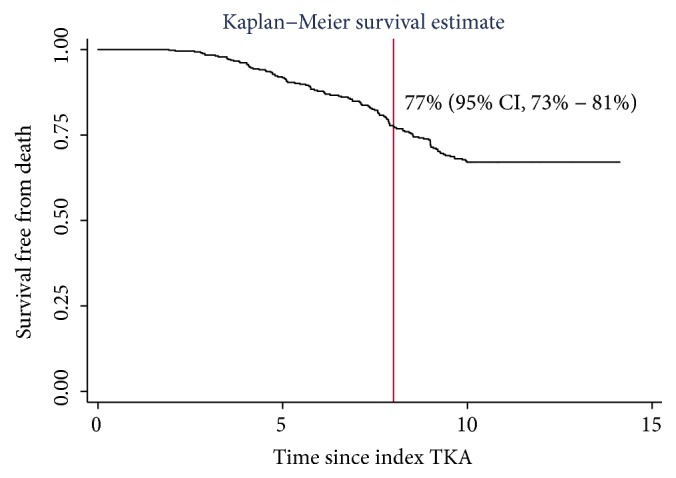
A Kaplan–Meier survival curve with death as failure.

The cumulative incidence of revision of any component was approximately 1% at a mean 8-year follow-up (range: 0.11–14.1 years) when accounting for the competing risk of death (Figure 3). Those who failed prior to two years remained in the analysis. When evaluating the worst-case scenario, considering all those lost to follow-up as failures, the cumulative incidence of failure at 8 years was approximately 10% (Figure 4). Finally, when reviewing only death as failure, the survivorship at 8 years was 77% (Figure 5).

There was a total of 28 complications for an incidence of 6%. Of the complication (Table 2), the majority consisted of adhesions (15/28, 53%), persistent pain (2/28, 7%), instability (2/28, 7%), and deep infections (2/28, 7%). All those with adhesions required a return to the operating theatre for manipulation under anesthesia (MUA). The median time to MUA was 6 weeks (IQR: 5–9 weeks). Nine patients (2%) required removal of one or more implant components over the entire follow-up period. The primary reasons for revision (Table 3) included persistent pain (2/9, 22%) and instability (2/9, 22%).

Of the 451 TKAs, 406 had a minimum two-year radiograph available for review. The mean valgus angle was 5 degrees (95% CI, 4.6–5.1). There were 18 TKAs (4.5%) with femoral radiolucencies at the bone interface, 2 (<1%) at the tibial bone interface on lateral views, five (1.2%) with A/P tibial bone interface lucencies, and 3 (<1%) with A/P tibial cement/prosthesis lucencies (Table 4). There was no evidence of subsidence at the last follow-up. The majority of radiolucencies were found in the femoral bone interface zone 1 (Tables 5–7). The zone with the greatest width of radiolucent lines was the femoral bone interface in femoral zone 4 (Table 8), whereas the width of radiolucent lines in the tibia was greatest in the bone interface of tibial zone 1 (Table 9).

The KS knee scores improved from an adjusted mean value of 41.6 (95% CI 39.2–44.0) preoperatively to 80.8 (95% CI, 78.5–83.1) postoperatively (p<0.001). Similarly, the KS functional score improved from a preoperative adjusted mean score of 42.6 (95% CI, 40.4–44.9) to a postoperative score of 71.0 (95% CI, 68.8–73.3, p<0.001). Finally, the arc of motion improved from a preoperative adjust mean value of 110° (95% CI, 108°–111°) to a postoperative value of 122° (95% CI, 120°–123°, p<0.001).

### 3.1. Subanalysis

Given the 26% mortality, the subanalysis for 8-year survival free from death was 77% (Figure 5). When dichotomized by age, 15% (67/451) of knees were from octogenarians and a greater proportion of them expired during the study time period (p<0.001), where 8% (27/335) of TKA patients less than eighty and 34.5% (40/116) of TKA patients ≥80 expired. The mean time to death was 6.6 ± 2 years.

## 4. Discussion

The gap balancing technique for total knee arthroplasty has been shown to be an effective method of performing TKA resulting in excellent clinical outcomes [20–22]. The most important finding in this study was that this posterior stabilized implant, with the gap balancing technique for obtaining symmetrical flexion and extension gaps, resulted in excellent survivorship and good clinical outcomes at a mean 8-year follow-up after TKA.

This study is not without limitations including that of missing data, a large number of deceased patients, and a potential for individual scientific bias. As the missing data was relatively low, approximately 11%, statistical methods were employed to impute this data appropriately. The mortality in this population was rather high for a primary TKA population with our data showing 8-year survival free from death at 77% (Figure 5), whereas a recent report using the National Joint Registry of England and Wales showed 8-year survival free from death at approximately 89% following primary TKA [23]. To better understand our mortality rate, we performed a subgroup analysis dichotomizing age into those ≥80 (octogenarians) and those <80. Fifteen percent (67/451) of knees were from octogenarians and approximately 35% of them had expired during the review (p<0.001), compared to only 8% of those <80. Murphy et al. [24] demonstrated increased mortality in patients greater than 80 years. As such, the risk for increased mortality in this population is likely due to the older nature of this cohort. Regardless, a competing risks model was used in order to determine the cumulative incidence of failure while accounting for those who expired making them unable to be at risk for failure. Finally, the two surgeons (MHB, EMM) have significant financial relationships with the manufacturer of the BKS system and as such the results of this study should be reviewed with this in mind.

Total knee arthroplasty has become one of the most reliable and durable procedures for restoring patient function. Excellent long-term results have been reported in the literature with survival rates ranging from 90 to 100 percent after 5–15 years, based upon failure definition [25–29]. The technique used with this system of gap balancing reliably provides a well-functioning TKA with results comparable to those previously reported in the literature [29]. Through a series of initial bone cuts, followed by soft tissue balancing, and then equalization of the flexion/extension gaps with final bone cuts, our cumulative incidence of failure was limited to 1%. Even with counting all patients lost to follow-up as knee failures (an extreme example), this TKA system demonstrated an acceptable cumulative incidence rate of failure (10%) at 8-year follow-up.

Of the 451 TKAs available for follow-up, 6% (n= 27) experience postoperative complications. The patellar fracture, the patellar tendon rupture, and the periprosthetic fracture all occurred postoperatively from traumatic falls. Both patients with “instability” did well with placement of a larger polyethylene spacer. The 15 patients (3.3%) with “adhesions” did well with a manipulation under anesthesia performed at a median of 5 weeks after the initial procedure. One patient underwent MUA 46 weeks after the index TKA due to continued adhesions and lack of compliance with his recovery pathway. This patient also struggled with mental health and was being treated for profound clinical depression. Unfortunately, he continued to ignore protocol and at last follow-up was still struggling with pain and lack of motion. The single case of tibial loosening was a 47-year-old male with evidence of polyethylene wear approximately 14 years after the primary TKA. At the time of revision surgery, a significant cyst was also noted on the femur and thus all components were revised.

Nonprogressive bone-cement lucencies were most commonly seen in femoral zone 1 and tibial zone 1. These were seen in the immediate postoperative X-rays. It is felt that these changes are seen merely as a result of the tangential cementing forces that occur with sliding the femoral component into place (zones 1 and 4 on the femoral side) as well as the often more sclerotic medial side (varus knees) with cementing of the tibial side. Again, these changes were seen early and had not progressed throughout follow-up.

Others have reported similar findings [30–32].

Regarding patient reported outcomes, our results are similar to prior reports where mean KS outcome scores at two- and five-year follow-up have ranged from 82.4 to 95.8 for the KS knee score and from 58.8 to 96.2 for the KS function score [33]. Both the KS knee score and functional scores achieved the MCID of approximately 6 points as reported by Lee et al. [13]. Finally, the arc of motion of a mean of 122 degrees postoperatively is also similar to that previously reported in the literature. While a functional ROM has been reported to be 105 degrees, we find that most patients will obtain between 120 and 130 degrees of flexion. Most flexion is limited by posterior soft tissue mass rather than limitations in the prosthesis. Slender patients will oftentimes exceed 140 degrees. Posterior stabilized knees allow for greater flexion through posterior femoral rollback than posterior cruciate retaining knees. The equalization of flexion/extension gaps along with soft tissue balancing also ensures the likelihood of an excellent resultant arc of motion.

## 5. Conclusion

Based on the results of the current study, a posterior stabilized primary TKA, implanted using a flexion and extension gap balancing technique, had excellent survivorship and outcomes at a mean 8-year follow-up. This technique allows all surgeons to obtain excellent results by following a step-by-step sequence that ensures equalization of the flexion/extension gaps as well as soft tissue balancing. Further studies are needed to evaluate the long-term survival of these implants.

## Figures and Tables

**Table 1:** Patient Characteristics.

Characteristics	Results
Sex, n (%)	
Female	330 (73%)
Male	122 (27%)

Age, mean (range)	69 (40 – 94)

BMI, mean (range)	30.2 (17.5 – 60.4)

ASA Score, median (IQR)	2 (2 – 3)

Deceased, n (%)	116 (26%)

Primary Diagnosis, n (%)	
Osteoarthritis	428 (95%)
Post-traumatic arthritis	8 (2%)
Rheumatoid arthritis	11 (2%)
Avascular necrosis	1 (<1%)
Psoriatic Arthritis	2 (<1%)
Other	1 (<1%)
Missing	1 (<1%)

**Table 2:** Postoperative complications.

Diagnosis	n (%)
Adhesions	15 (53%)

Persistent pain	2 (7%)

Instability	2 (7%)

Superficial infections	2 (7%)

Patellar fracture	2 (7%)

Deep infections	1 (4%)

Missing	1 (4%)

Patellar tendon rupture	1 (4%)

Tibial loosening	1 (4%)

Periprosthetic femur fracture	1 (4%)

**Table 3:** Reasons for Revision (removal of one or more implant components).

Diagnosis	n (%)
Persistent pain	2 (22%)

Instability	2 (22%)

Deep infection	1 (11%)

Tibial Loosening	1 (11%)

Periprosthetic fracture	1 (11%)

Patellar Tendon Rupture	1 (11%)

Unknown	1 (11%)

**Table 4:** Distribution of radiolucent lines.

Component	Patients with Radiolucencies, n (%)
Femoral: bone interface	18 (4.5%)

Femoral: cement/prosthesis	2 (<1%)

Patella: bone interface	0

Patella: cement/prosthesis	0

Tibia: bone interface (lateral)	2 (<1%)

Tibia: cement/prosthesis (lateral)	0

Tibia: bone interface (AP^a^)	5 (1.2%)

Tibia: cement/prosthesis (AP^a^)	3 (<1%)

a: anteroposterior.

**Table 5:** Frequency of radiolucent lines in the femoral zones.

Radiolucent Zones	n (%)
Femoral Bone	
zone 1	14 (78%)
zone 2	0
zone 3	0
zone 4	6 (33%)
zone 5	0
zone 6	0
zone 7	0

Femoral cement/prosthesis	
zone 1	2 (100%)
zone 2	0
zone 3	0
zone 4	0
zone 5	0
zone 6	0
zone 7	0

**Table 6:** Frequency of radiolucent lines in the anteroposterior (AP) tibial zones.

Radiolucent Zones	n (%)
Tibia: bone interface (AP^a^)	
zone 1	4 (80)
zone 2	1 (20)
zone 3	1 (20)
zone 4	1 (20)
zone 5	1 (20)
zone 6	1 (20)
zone 7	0

Tibial: cement/prosthesis (AP^a^)	
zone 1	2 (67)
zone 2	2 (67)
zone 3	2 (67)
zone 4	2 (67)
zone 5	0
zone 6	0
zone 7	0

a: anteroposterior.

**Table 7:** Frequency of radiolucent lines in the Lateral Tibial zones.

Radiolucent Zones	n (%)
Tibia: bone interface (lateral)	
zone 1	1 (50%)
zone 2	0
zone 3	1 (50%)

Radiolucent Zones	n (%)

Tibia: bone interface (lateral)	
zone 1	1 (50%)
zone 2	0
zone 3	1 (50%)

**Table 8:** The proportion of radiolucent line widths per femoral zone.

Component	None	<1 mm	1 -2 mm	>2 mm
Femoral: Bone interface, n=18				
zone 1	4	5	9	0
zone 4	12	6	0	0

Femoral: cement/prosthesis, n=2				
zone 1	2	0	0	0

Component	None	<1 mm	1 -2 mm	>2 mm

Femoral: Bone interface, n=18				
zone 1	4	5	9	0
zone 4	12	6	0	0

Femoral: cement/prosthesis, n=2				
zone 1	2	0	0	0

**Table 9:** The proportion of radiolucent line widths per tibial zone.

Component	None	<1 mm	1 -2 mm	>2 mm
Tibia: bone interface (AP^a^), n=5	1	1	3	0
zone 1	4	1	0	0
zone 2	4	1	0	0
zone 3	4	1	0	0
zone 4	4	1	0	0
zone 5	1	1	0	0
zone 6				

Tibia: bone cement/prosthesis (AP^a^), n=3	1	1	0	1
zone 1	1	1	0	1
zone 2	1	2	0	0
zone 3	1	2	0	0
zone 4	3	0	0	0
zone 5	3	0	0	0
zone 6				

a: anteroposterior.

## Data Availability

The data used to support the findings of this study are available from the corresponding author upon request.
